# The role of LCN2 and LCN2-MMP9 in spondylitis radiographic development: gender and HLA-B27 status differences

**DOI:** 10.1186/s13075-022-02854-2

**Published:** 2022-07-08

**Authors:** Florence W. L. Tsui, Aifeng Lin, Ismail Sari, Zhenbo Zhang, Kenneth P. H. Pritzker, Hing Wo Tsui, Robert D. Inman

**Affiliations:** 1 Department of Immunology, University of Toronto, Toronto, Ontario Canada; 2 KeyIntel Medical Inc., Toronto, Ontario Canada; 3 Krembil Research Institute, University Health Network, Toronto, Ontario Canada; 4 Schroeder Arthritis Institute, University Health Network, Toronto, Ontario Canada; 5 Department of Internal Medicine, Dokuz Eylul University, Faulty of Medicine, Izmir, Turkey; 6 Ontario Institute for Cancer Research, Toronto, Ontario Canada; 7 Department of Laboratory Medicine and Pathobiology, University of Toronto, Toronto, Canada; 8 Department of Medicine and Institute of Medical Sciences, University of Toronto, Toronto, Ontario Canada

**Keywords:** Axial spondyloarthritis, Lipocalin 2, Lipocalin 2-MMP9 complex, MRI, SIJ and spinal inflammation, mSASSS, Gender differences, HLA-B27 effect

## Abstract

**Background:**

Male HLA-B27-positive radiographic-axial spondyloarthritis (r-axSpA) patients are prone to have severe spinal radiographic progression, but the underlying mechanisms are unclear. We recently showed that persistently elevated Lipocalin 2 (LCN2; L) reflects sacroiliac joint (SIJ) inflammation. LCN2 binds to MMP9. Concomitant elevation of L and LCN2-MMP9 (LM) was detected in many inflammatory diseases. We asked whether L and LM play similar roles in r-axSpA pathogenesis.

**Methods:**

We analyzed 190 axSpA patients (123 radiographic and 67 non-radiographic axSpA) who had no detectable circulating Oncostatin M, to avoid complications due to cross-talk between pathways. L and LM levels from a single blood sample of each patient were measured and were correlated with MRI and modified stoke AS (mSASS) scoring. Association of elevated L (L+) or concurrent L+ and elevated LM (LM+) patterns with B27 status and gender were assessed.

**Results:**

In L+LM+ axSpA patients, both L and LM levels correlated with MRI SPARCC SIJ scores, but only LM levels correlated with MRI Berlin Spine Scores, suggesting LM is a biomarker for both SIJ and spinal inflammation. Among patients with minimal spinal ankylosis (mSASSS < 10), 65% of male r-axSpA patients are L+LM+, while 30% and 64% of female patients are L+LM+ and L+, respectively, supporting the role of LM with disease progression. In B27+ L+LM+ male patients, both L and LM (but not CRP) levels correlate with mSASSS. B27 positivity and maleness have additive effects on spondylitis progression, suggesting concurrent high L and LM elevations are associated with B27+ male patients having more significant radiographic damage. L+ B27-negative male patients or L+ female patients are more likely to have milder disease.

**Conclusion:**

L and LM are informative biomarkers for SIJ and spinal inflammation, as well as for ankylosing development in r-axSpA patients. Distinctive L+LM+ or L+ patterns not only could distinguish clinically aggressive vs milder course of disease, respectively, but also provide an explanation for B27-positive male patients being the most susceptible to severe ankylosis.

**Supplementary Information:**

The online version contains supplementary material available at 10.1186/s13075-022-02854-2.

## Background

There are at least two key factors to ankylosis progression in radiographic axial spondyloarthritis (r-axSpA or ankylosing spondylitis (AS)), a progressive debilitating disease: gender and HLA-B27 status.

More men than women have AS (3:1 ratio). Higher radiographic progression is present in men (45% vs 33%) [1]. Genetic studies have not reliably shown an association of HLA-B27 positivity with r-axSpA spinal disease progression. However, HLA-B27-positive (B27+) r-axSpA patients have more radiographic damage than those who are B27-negative (B27−) [2]. The mechanistic differences leading to more B27+ male AS patients with fused spines are unclear. In our earlier studies, we showed that two ossification-related genes, *ANKH* and *TNAP*, have gender differences in the susceptibility loci [3, 4]. Recently, using *ank/ank* mice with a fused spine, we identified the LCN2-associated pathway and showed that higher LCN2 levels correlate with more severe ankylosis in AS patients [5]. In our more recent report, we showed that LCN2, an acute phase protein (APP), is elevated in about 70% of axSpA patients in our cohort (*n* = 286) [6]. Profiling of treatment responses showed that the outcomes were not affected by cofactors such as B27 status, gender, and comorbidities. Our interpretation is that axSpA patients have common early pathogenic events. Heterogeneity in the subsequent clinical course of the disease is influenced by cofactors such as gender and B27 status. In an attempt to identify one of the sequences of downstream events, we asked whether LCN2-MMP9 complexes (LM) also play a role in AS pathogenesis involving the LCN2-associated pathway.

LCN2 binds to MMP9, forming a complex (LM) and protecting MMP9 from degradation. When complexed with LCN2, MMP9 has increased enzymatic activity in vitro [7]. MMP9 is expressed in inflammatory cells and osteoclasts and thus could influence skeletal cell differentiation via regulating the inflammatory response and the distribution of inflammatory cells, under different mechanical stimuli during bone repair [8].

Similar to LCN2, the elevation of LM has been reported in many inflammatory and rheumatic diseases. In community-acquired pneumonia (CAP), LM has a significant correlation with the level of LCN2 and is directly proportional to LCN2 levels [9]. Concomitant elevations of LCN2 (L) and LCN2-MMP9 (LM) were detected in smokers with chronic obstructive pulmonary disease (COPD). However, LM, but not L, can discriminate between smokers with and without COPD [10]. In both Crohn’s disease (CD) and ulcerative colitis (UC), LM, but not L, is a surrogate marker of endoscopic and histological mucosal healing after treatment with infliximab [11, 12]. Thus, a direct correlation of L vs LM might not exist in some diseases, implying that L and LM could have different roles in the disease process.

In this study, we explore whether L and LM have similar or differential roles in different aspects of AS pathogenesis (joint inflammation and ankylosis).

## Methods

### Patients

We previously showed that 69% (197/286) of axSpA patients have elevated LCN2 levels, and 62% (123/197) of them have single LCN2 pathway involvement [6]. In this report, we focus on assessing this group of axSpA patients having elevated LCN2 levels but no detectable OSM levels. This is mainly for minimizing complexity in data interpretation as we have some indications that there is an interaction between the LCN2-associated and the OSM pathways. For analyzing this subgroup of OSM-negative patients, L and LM levels from the same blood sample in 190 axSpA patients (123 radiographic and 67 non-radiographic axSpA) were measured. Different groups/subgroups of axSpA patients were used in separate assessments as specified in the “Results” section. All patients assessed had serum banking and concurrent clinical parameters. Additional file 1: Table S1 summarizes the demographic features of this cohort.

### Study approval

The study was approved by the University Health Network (UHN) research ethics committee. All participating patients provided written informed consent, which was received from participants prior to inclusion in the study. Participants were identified by study number in the analyses.

### MRI and radiographic damage scoring

Among patients who had MRI assessments, 29 of them were used for association analysis. Spondyloarthritis Research Consortium of Canada (SPARCC) scoring and Berlin spinal joint scoring [13–15] were evaluated. Scoring was done independently by two readers (IS and SL). The mean scores were used for the correlation analysis with L or LM levels (Pearson’s correlation and Spearman’s rho [non-parametric] correlation). MRI taken within 12 months of the time of biomarker measurements were used for this analysis.

Radiographic damage was assessed by the Modified Stoke Ankylosing Spondylitis Spinal Score (mSASSS) [16]. mSASSS at the time of biomarker measurements was used for the correlation analysis. Comparisons of biomarkers levels were made between 54 male and 22 female r-axSpA patients with mSASSS < 10. For the correlation analysis of B27+ male r-axSpA patients, 32 with mSASSS > 11 and 16 with mSASSS < 10 were used.

### Quantification of serum LCN2, LCN2-MMP9, and OSM levels

The samples (stored at − 70 °C) from each patient were thawed and analyzed at the same time to minimize assessment variabilities. L and LM levels were measured by sandwich ELISA according to the manufacturer’s protocol (LCN2 ELISA kit: R & D Systems, DLCN20; human MMP9/NGAL complex kit: R & D Systems, DY8556). The mean minimum detectable limit for human LCN2 and LCN2-MMP9 was 0.012 ng/ml and 0.013 ng/ml, respectively (R & D Systems). The LCN2 kit showed no cross-reactivity with human MMP9 and does not measure LCN2 hetero-complexes. The LCN2-MMP9 kit exhibited no cross-reactivity with LCN2 monomers or homodimers. Previously, we used 100 ng/ml as the cutoff for LCN2 (as determined by mean + 2SD of healthy controls) [5]. In a subsequent report [6] and this report, we used 150 ng/ml to increase specificity and positive predictive value. The cutoff for LM is 100 ng/ml.

OSM levels were measured by ELISA according to the manufacturer’s protocol (Thermo Scientific, EHOSM). The lower limit of detection for human OSM was 1 pg/ml (Thermo Scientific). Patients with undetectable OSM levels were used in this study.

### Statistics

One-way analysis of variance (ANOVA) and Student’s *t*-test were carried out using the GraphPad Prism program. A *p*-value of less than 0.05 was considered significant. Data are presented as mean ± standard error. Calculators from socsistatistics.com were used for the chi-square tests, Pearson’s correlation coefficient, and Spearman’s rho correlation.

## Results

### axSpA patients have elevated LCN2 (L) and LCN2-MMP9 (LM) levels

**Fig. 1:**
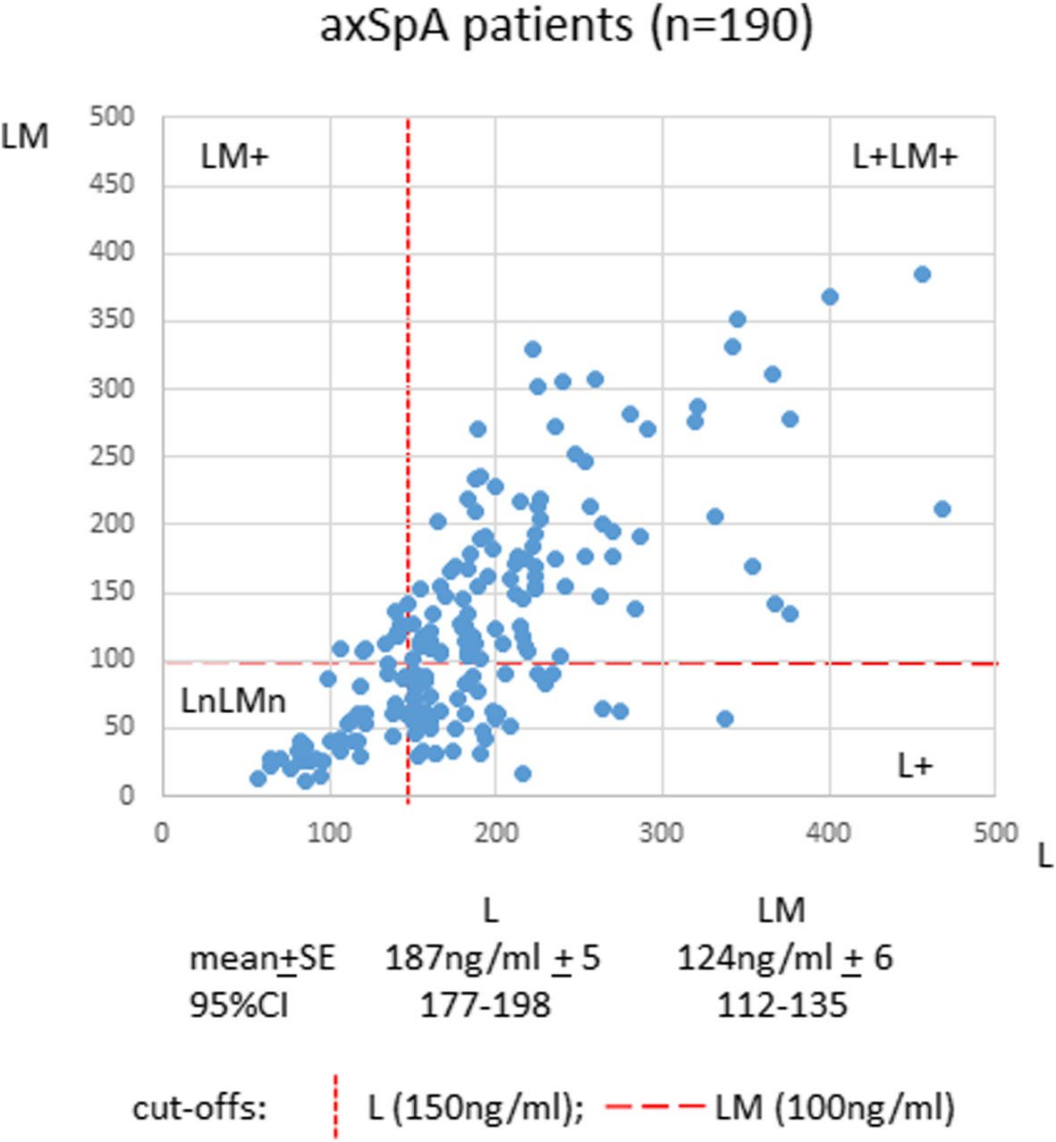
LCN2 (L) and LCN2-MMP9 (LM) levels in axSpA patients with no detectable OSM. L (*x*-axis) and LM (*y*-axis) levels were measured in a single blood sample from each of the 190 axSpA patients. There are 3 patterns of L or/and LM elevation: L+ (22%), LM+ (6%), and L+LM+ (52%)

We previously reported that axSpA patients have pathological involvement of two pathways (LCN2-associated and OSM), acting singly or in combination [6]. About 60% of axSpA patients have LCN2 involvement only. This study is focused on analyzing this subgroup with undetectable OSM in the circulation. We compared the L vs LM levels from a single blood sample in 190 axSpA patients (123 radiographic and 67 non-radiographic axSpA; Fig. 1). The cutoffs for L and LM are 150 ng/ml and 100 ng/ml, respectively. Overall, the L and LM levels of this subgroup are 187 ng/ml ± 5 and 124 ng ± 6, respectively. Fifty-two percent (98/190) of the patients are L+LM+ concurrently. Twenty-two percent (42/190) are L+, but with normal LM levels. Six percent (11/190) are LM+, but with normal L levels. Twenty-one percent (39/190) have normal levels of both L and LM (LnLMn).

### Correlation of LM levels with MRI scores in axSpA patients

**Fig. 2:**
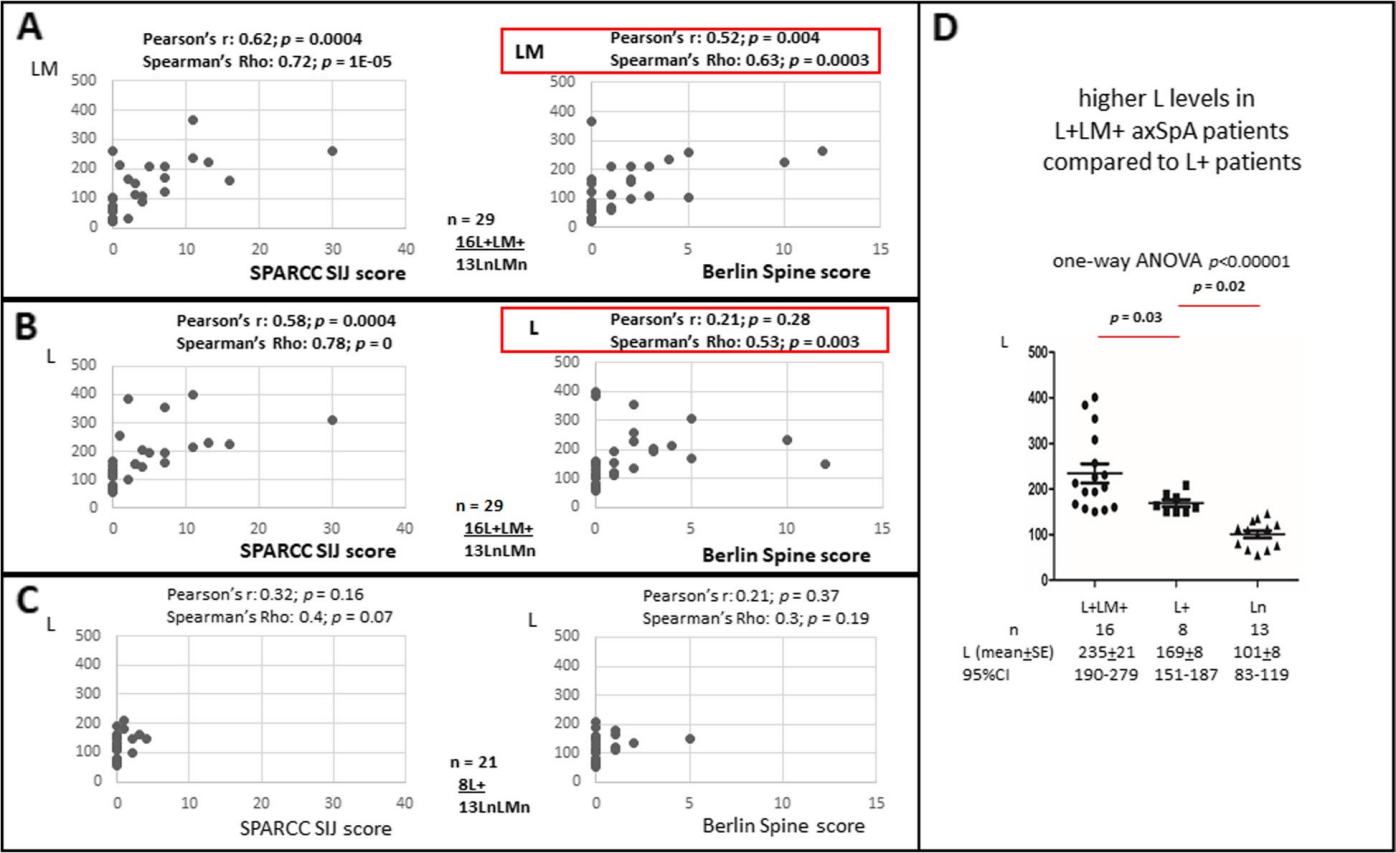
Correlation of LCN2 (L) or LCN2-MMP9 (LM) levels with MRI scores. **A** Correlation of LM levels with SPARCC SIJ scores and Berlin Spine Scores in patients with elevation of both L and LM levels (L+LM+; *n* = 16) vs patients with normal L and LM levels (LnLMn; *n* = 13). **B** Correlation of L levels with SPARCC SIJ scores and Berlin Spine Scores in L+LM+ vs LnLMn patients. **C** Correlation of L levels with SPARCC SIJ scores and Berlin Spine Scores in L+ (*n* = 8) vs LnLMn (*n* = 13) patients. Pearson’s correlation coefficient test and Spearman’s rho correlation calculation were used to determine the significance. **D** L levels in L+LM+ patients are significantly higher than that in L+ patients. One-way analysis of variance (ANOVA) including Tukey honestly significant difference (HSD) was used to determine the significance

We showed previously that elevated L levels are correlated significantly with MRI SPARCC SIJ scores in axSpA patients [6]. In this study, we ask whether elevations of both L and LM levels (L+LM+) reflect joint inflammation in axSpA patients. We compared L and LM levels in L+LM+ axSpA patients (*n* = 16) vs patients with normal L and LM levels (LnLMn; *n* = 13) with MRI SPARCC SIJ and Berlin Spine Scores. Both L and LM levels correlate significantly with MRI SPARCC SIJ scores. In both cases, *p*-values of Pearson’s *r* are 0.0004 (Pearson’s *r* 0.62 for LM and 0.58 for L; Fig. 2A, B). For correlation with MRI Berlin Spinal Scores, significant correlation is found with LM but not L levels, using Pearson’s *r* correlation (*p*-value = 0.004 for LM levels [Pearson’s *r* 0.52] and *p-*value = 0.28 for L levels [Pearson’s *r* 0.21]; Fig. 2A for LM correlations and 2B for L correlations). However, using non-parametric Spearman’s rho correlation, both LM and L levels had a significant correlation with MRI Berlin Spinal Scores. The correlation is more robust with LM levels (Spearman’s rho 0.63; *p*-value = 0.0003) vs 0.003 for L levels (Spearman’s rho 0.53; *p* value = 0.003; Fig. 2A, B).

As controls, we asked whether there is any correlation of L levels in patients with elevated L levels (L+; *n* = 8) compared to patients with normal L and LM levels (LnLMn; *n* = 13). No correlation is found in this comparison for both MRI SPARCC SIJ scores and MRI Berlin Spinal Scores (Fig. 2C). L+ patients have lower L levels (169 ng/ml ± 8) compared to L+LM+ patients (235 ng/ml ± 21; *p* = 0.03; Fig. 2D). In addition, L+ patients have low MRI scores and thus might explain why no correlation is found.

We have too few LM+ patients (< 3) with MRI scores to address similar correlations. Taken together, higher L and LM levels in L+LM+ patients are associated with SIJ and spinal inflammation. This observation reflects that L+LM+ patients have more severe SIJ and spinal inflammation.

### Gender differences in r-axSpA patients with minimal spinal ankylosis (mSASSS < 10)

**Fig. 3:**
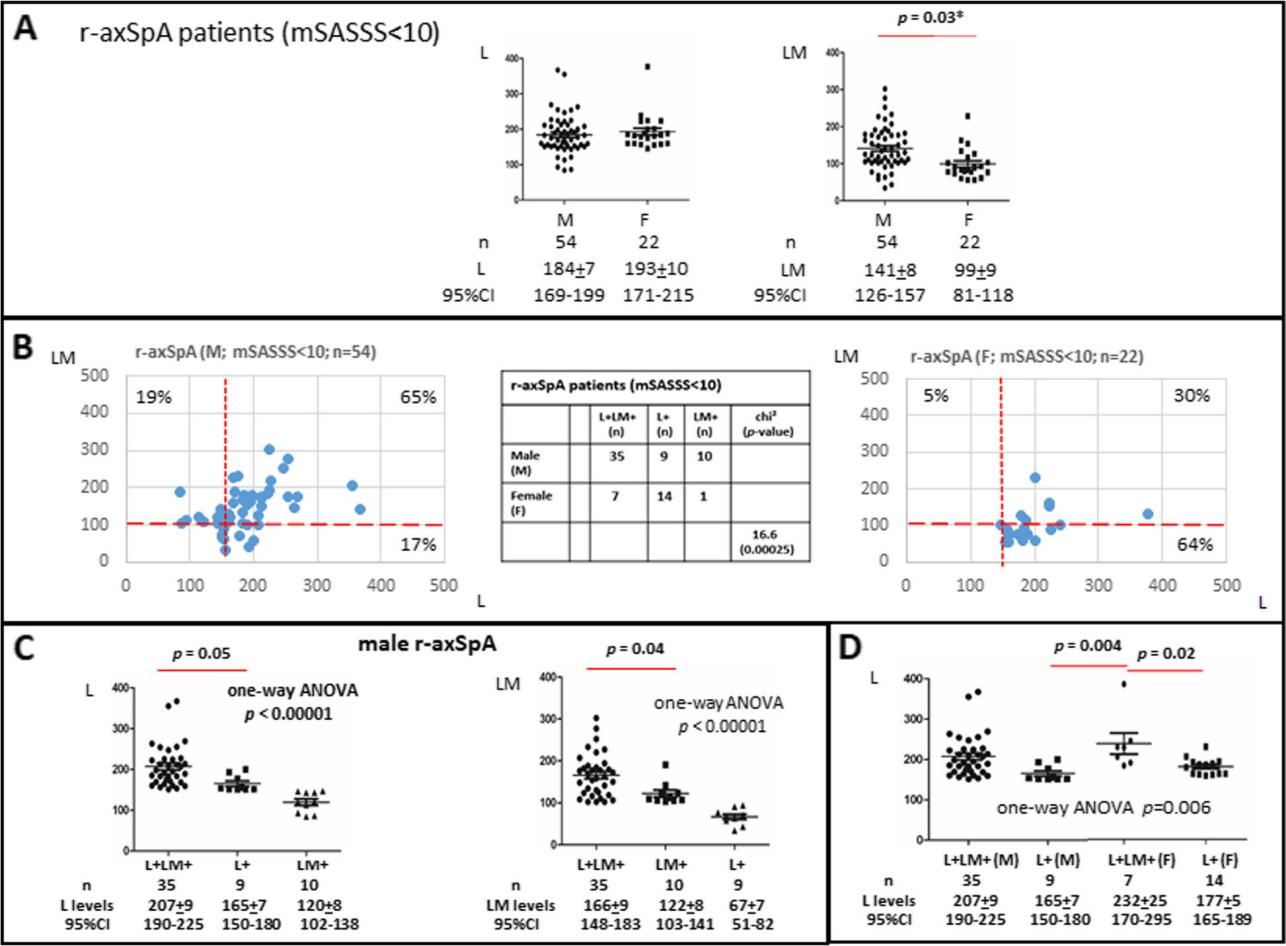
Gender differences in L+ and L+M+ patterns in r-axSpA patients with mSASSS < 10. **A** Female patients (*n* = 22) have significantly lower LM levels compared to male patients (*n* = 54). **B** Pattern profiling of male vs female patients showed significant differences. Sixty-five percent of male patients are L+LM+, and 64% of female patients are L+. The chi^2^ test was used to determine the significance. **C** Significant quantitative difference in L/LM levels on comparing L levels in L+LM+ vs L+ male patients and LM levels in L+LM+ vs LM+ male patients. **D** L levels in L+LM+ female patients are higher than in L+ patients (male and female). One-way analysis of variance (ANOVA) including Tukey honestly significant difference (HSD) was used to determine the significance

One of the aims of this study is to assess the role of LM in joint inflammation. As we have more r-axSpA patients in our cohort, we selected a subgroup of these patients with minimal ankylosis to avoid complication of data interpretation for distinguishing the potential role of LM in ankylosis. While male (M) and female (F) r-axSpA patients have similarly elevated L levels (184 ng/ml ± 7 vs 193 ng/ml ± 10, M [*n* = 54] vs F [*n* = 22], respectively), female patients have significantly lower LM levels compared to male patients (141 ng/ml ± 8 for male vs 99 ng/ml ± 9 for female patients; *p*-value = 0.03; Fig. 3A).

Profiling of the prevalence of L+, LM+ L+LM+ patients also revealed gender differences. Sixty-five percent of male patients (35/54) are L+LM+, while 64% (14/22) female patients are L+ (chi^2^ = 16.6, *p*-value = 0.00025; Fig. 3B). Higher L and LM levels are found in male r-axSpA L+LM+ patients with no ankylosis, compared to L+ or LM+ male patients (Fig. 3C). L levels in L+LM+ patients (*n* = 35) are 207 ng/ml ± 9. This is significantly higher than the L levels in L+ patients (*n* = 9; 165 ng/ml ± 7; *p* = 0.05; Fig. 3C). Similarly, higher LM levels are present in L+LM+ patients (*n* = 35, 166 ng/ml ± 9) vs LM+ patients (*n* =10; 122 ng/ml ± 8; *p* = 0.04; Fig. 3C). In female patients, there is also significant L level differences in L+LM+ patients (*n* = 7; 232 ng/ml ± 25) vs L+ patients (*n* = 14; 177 ng/ml ± 5; *p* = 0.02; Fig. 3D). The L levels in female L+LM+ patients are also higher than in male L+ patients (232 ng/ml ± 25 vs 165 ng/ml ± 7, respectively; *p* = 0.004; Fig. 3D).

In view of this gender difference of 65% vs 30% L+LM+ in male vs female patients, respectively, and 17% vs 64% L+ in male vs female patients, respectively, we hypothesize that concurrently higher L+ and LM+ levels might enhance spinal ankylosis progression. As female patients have lower LM levels, this might explain a long-time clinical observation that there are fewer female patients with spinal ankylosis compared to male patients [17].

### B27 positivity-related differences in r-axSpA male patients

Aside from the observation that spinal ankylosis is more prevalent in male r-axSpA patients compared to female patients [1], it is also known for decades that B27-positive (B27+) male patients have more severe spinal radiographic progression and worse spinal mobility [15]. Our cohort has too few female r-axSpA patients (less than 30) to assess this issue in details, and thus, the following study is focused on male r-axSpA patients. As we showed that both L and LM levels correlated to MRI SPARCC SIJ and Berlin Spine Scores, we asked whether elevated L/LM levels might also reflect spinal radiographic progression in male r-axSpA patients. For this analysis, we have 48 B27-positive (B27+) and 20 B27-negative (B27−) male r-axSpA patients; 67% (32/48) and 40% (8/20), respectively, have mSASSS > 11 (chi^2^ 4.1; *p* = 0.04).

There are significant differences in the elevated L vs LM profiles between B27+ and B27− patients. Higher L and LM levels are present in B27+ compared to B27− patients (B27+ vs B27−: L levels—242 ng/ml ± 10 vs 192 ng/ml ± 12, *p* = 0.002; LM levels—190 ng/ml ± 12 vs 102 ng/ml ± 13, *p* = 0.00005; Additional file 1: Fig. S1).

**Fig. 4:**
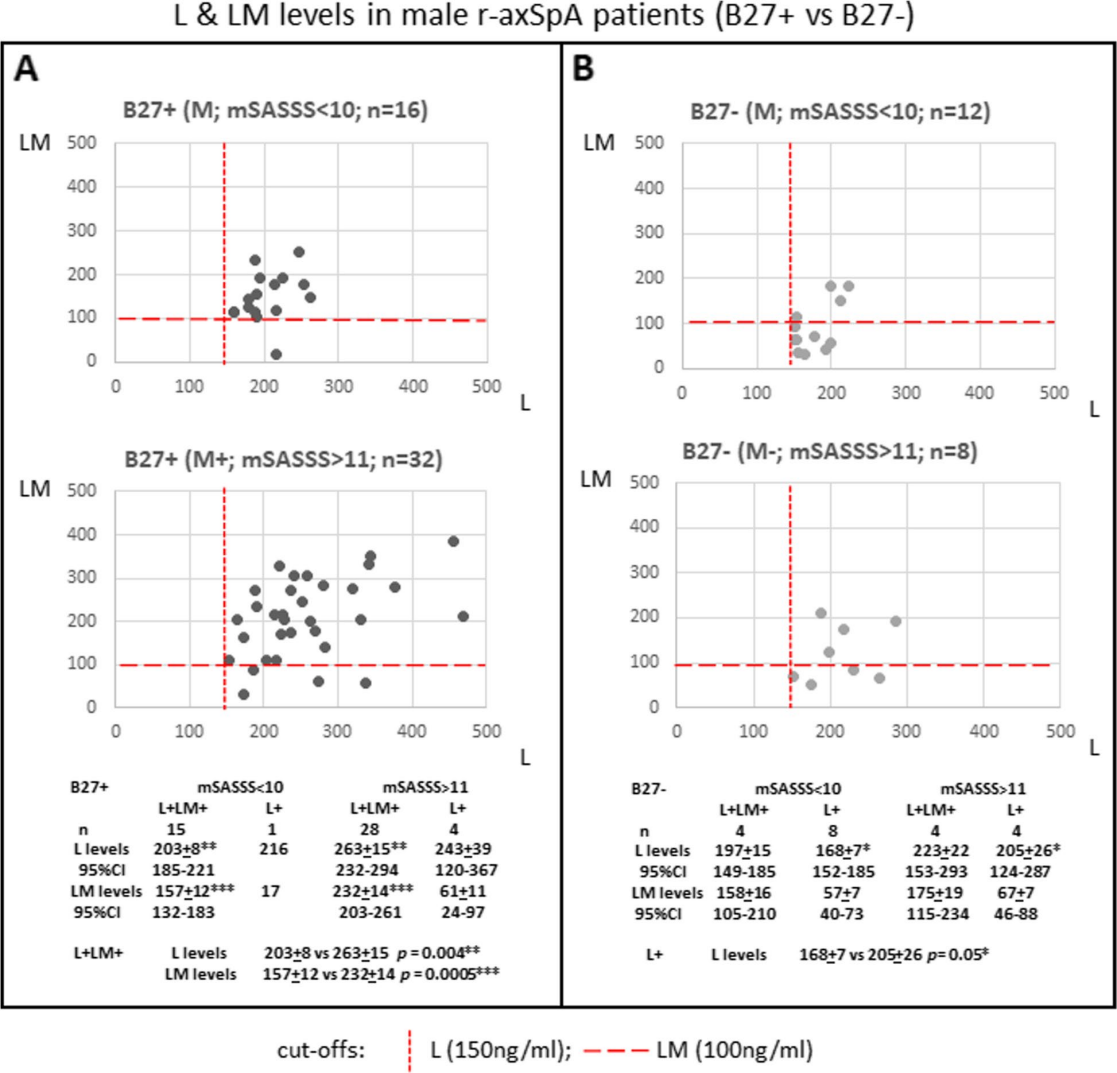
Comparison of L and LM levels in male B27+ vs B27− r-axSpA patients. **A** Plots of LM (*y*-axis) vs L (*x*-axis) levels in B27+ patients with mSASSS < 10 (upper graph) and B27+ patients with mSASSS > 11 (lower graph). B27+ L+LM+ patients with mSASSS > 11 have significantly higher L and LM levels compared to those with mSASSS < 10. **B** Plots of LM vs L levels in B27− patients with mSASSS < 10 (upper graph) and B27− patients with mSASSS > 11 (lower graph). B27-L+ patients with mSASSS > 11 have significantly higher L levels compared to those with mSASSS < 10. One-way analysis of variance (ANOVA) including Tukey honestly significant difference (HSD) was used to determine the significance

Profiling was carried out in B27+ vs B27− patients, both separately in two subgroups based on the status of spinal ankylosis (mSASSS < 10 vs mSASSS > 11). For B27+ patients, irrespective of spinal ankylosis status, most of them are L+LM+ patients (94% [15/16] vs 88% [28/32] for mSASSS < 10 vs mSASSS > 11 patients, respectively). In these two patient subgroups, significantly higher L and LM levels are present in mSASSS > 11 patients (L levels 263 ng/ml ± 15 vs 203 ng/ml ± 8, *p* = 0.004; LM levels 232 ng/ml ± 14 vs 157 ng/ml ± 12, *p* = 0.0005; Fig. 4A). In contrast, for B27− patients, irrespective of spinal ankylosis status, L+ patients are prevalent (67% [8/12] vs 50% [4/8] for mSASSS < 10 vs mSASSS > 11 patients, respectively). Elevations of L levels in mSASSS > 11 patients are higher than those in mSASSS < 10 patients (205 ng/ml ± 26 vs 168 ng/ml ± 7, *p* = 0.05; Fig. 4B).

Sixty-five percent (28/43) of L+LM+ B27+ male r-axSpA patients have higher L and LM levels when their mSASSS progressed to > 11. Compared to B27+ patients, the prevalence of B27− L+LM+ patients is lower (50%, 4/8), and the LM levels overlap with patients with minimal spinal ankylosis (mSASS < 10), suggesting indirectly that higher LM levels likely facilitate spinal radiographic progression.

### Correlation of L vs LM levels with spinal ankylosis in male r-axSpA patients via comparison of patients without and with spinal ankylosis

**Fig. 5:**
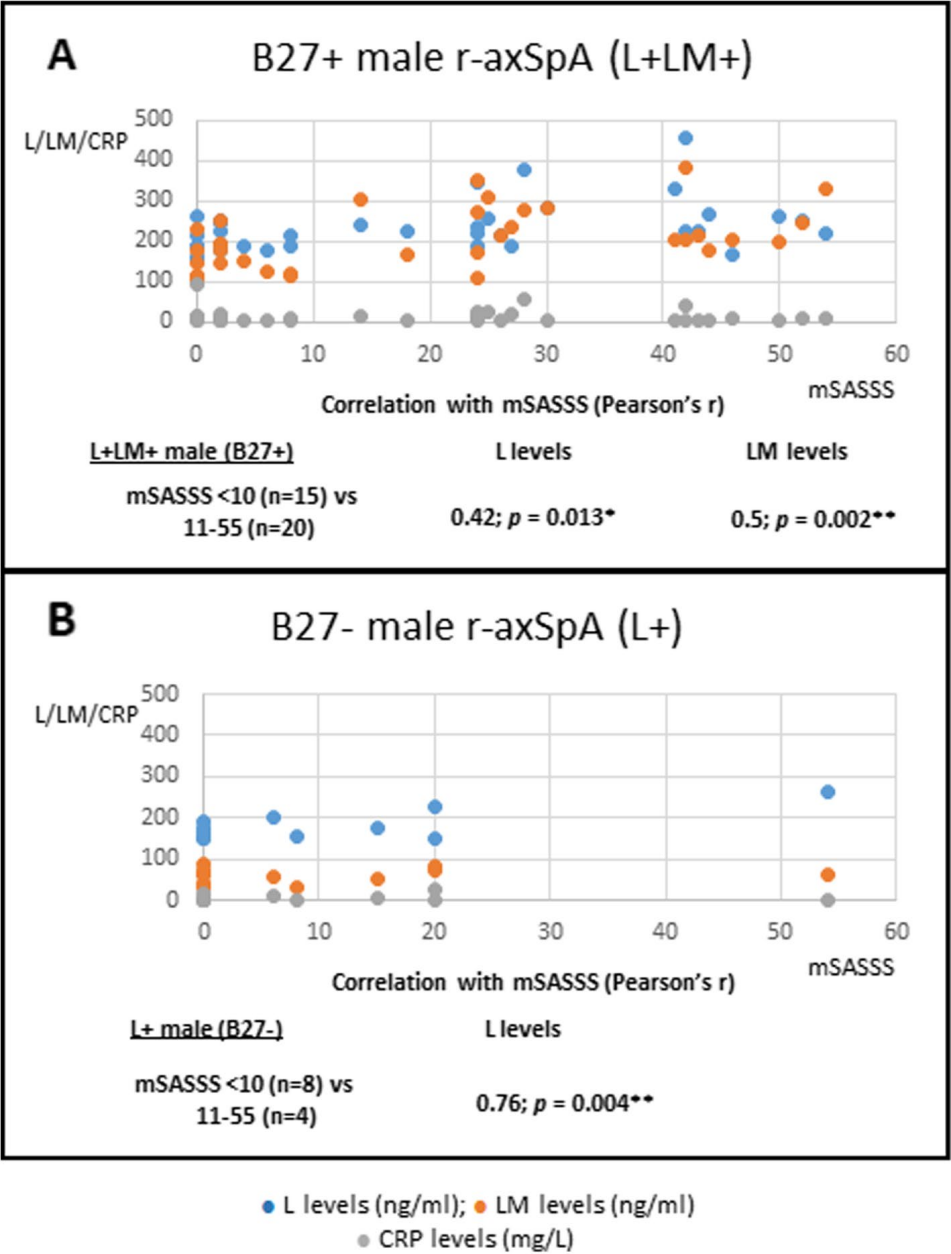
Correlation of L and LM levels with mSASS Scores in male r-axSpA patients. **A** correlation of L (blue dots) and LM (orange dots) levels with mSASSS in B27+ L+LM+ patients (mSASS 11–55; *n* = 20) vs those with mSASSS < 10 (*n* = 15). **B** Correlation of L (blue dots) and LM (orange dots) levels with male B27-L+ patients (mSASSS 11–55; *n* = 4) vs those with mSASSS < 10 (*n* = 8). In both **A** and **B**, there is no correlation of CRP (gray dots) with mSASSS. Pearson’s correlation coefficient tests were used to determine the significance

In an earlier section, we showed that in L+LM+ r-axSpA patients, a significant correlation of MRI Berlin Spine Score is robust with LM levels, implying that elevated LM levels reflect spinal joint inflammation. Thus, we asked whether LM levels correlate with spinal ankylosis in L+LM+ B27+ male r-axSpA patients. We compared 15 L+LM+B27+ mSASSS < 10 patients with 20 L+LM+B27+ patients with mSASSS 11–55. Both L and LM levels (but not CRP) correlate with mSASS Scores (Pearson’s *r* 0.42 for L [*p* = 0.013] and 0.5 for LM [*p* = 0.002]; Fig. 5A).

As we showed earlier that 60% (12/20; Fig. 4B) of B27− male r-axSpA patients are in the L+ category (i.e., with normal LM levels), we asked whether L levels in these patients correlate with mSASS Scores. Thirty-three percent (4/12) of L+ patients have mSASSS > 11, and their L levels correlate with mSASSS (Pearson’s *r* 0.76, *p* = 0.004; Fig. 5B). In this comparison, there is no correlation of CRP with mSASS Scores.

Taken together, the more severe spinal radiographic damage observed in male B27+ r-axSpA patients could be explained by the cumulative effects of both L+ and LM+ on spinal inflammation and ankylosis.

However, it is difficult to distinguish whether L+ and LM+ levels in r-axSpA patients with ankylosis truly reflect the degree of spinal ankylosis or merely reflect the extent of spinal inflammation at different stages of radiographic progression.

We asked whether there is an additive effect in B27 positivity and sex by comparing B27+ male vs B27+ female patients in their L/LM pattern profiles. For mSASSS < 10 male patients, 15 are L+LM+ and only one L+. For mSASSS > 11 male patients, there are 28 L+LM+ and 4 L+. In contrast, for mSASSS < 10 female patients, 5 of them are L+LM+ vs 6 L+. There are only one L+LM+ and one L+ female patients with mSASSS > 11. There is a significant gender difference in the profiles of these B27-positive patients (chi^2^ = 23, *p* < 0.00004; Table 1), suggesting an additive effect of B27 positivity and maleness. L+LM+ is the predominant pattern irrespective of the mSASSS status in the B27+ male r-axSpA patients, implicating that the additive effect of B27 positivity and maleness likely occurs prior to spinal ankylosis progression.

**Table 1:**
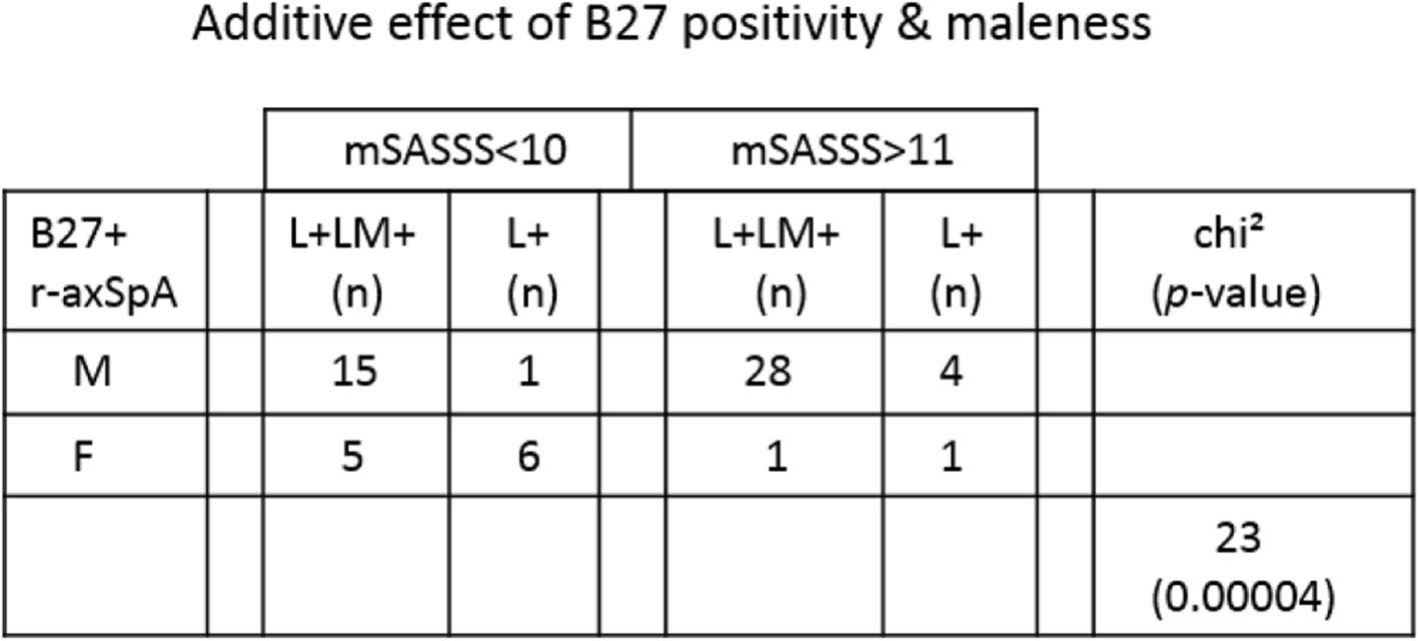
Additive effect of HLA-B27 positivity and maleness in r-axSpA patients. Comparison of the prevalence of L+LM+ vs L+ in B27+ male vs B27+ female patients (mSASSS < 10 and mSASSS > 11). About 90% of male patients vs 50% of female patients have the L+LM+ pattern, though all patients are B27+. Pearson’s chi-square test was used to determine the significance

**Fig. 6:**
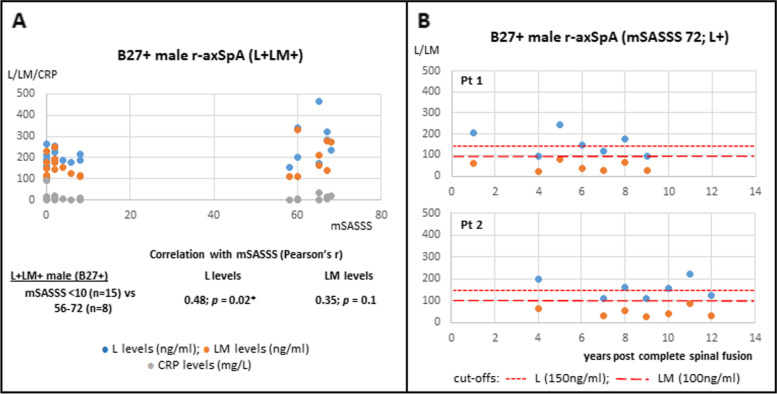
L and LM levels in B27+ male r-axSpA patients with severe spinal ankylosis. **A** Correlation of L (blue dots) and LM (orange dots) levels with mSASSS in B27+ L+LM+ patients (mSASSS 56–72; *n* = 8) vs those with mSASSS < 10 (*n* = 15). L but not LM levels were significantly correlated with mSASSS. Pearson’s correlation coefficient tests were used to determine the significance. **B** Sequential measurements of L (blue dots) and LM (orange dots) levels in two (Pt 1 and Pt 2) B27+ male patients with a completely fused spine (mSASSS 72)

As spinal inflammation could subside in patients with near fused spines (mSASSS 56–72), we compared 8 L+LM+B27+ patients with mSASSS 56–72 with 15 L+LM+B27+ mSASSS < 10 patients. L levels remain correlated with mSASSS (Pearson’ *r* 0.48, *p* = 0.02; Fig. 6A), but LM levels no longer correlate with mSASSS (Pearson’s *r* 0.35, *p* = 0.1; Fig. 6A). This observation implies that joint inflammation in patients with near fused spine is likely due to L but not LM elevation.

In our cohort, we have 2 male B27+ r-axSpA patients with a completely fused spine (mSASSS 72), and sequential L/LM measurements are available post-spinal fusion for up to 12 years. For both patients, during this long follow-up period, LM levels remain normal, but there were fluctuating elevations of L levels despite continual biologics treatments (Fig. 6B). This observation suggests that elevation of L exists throughout the disease course irrespective of ankylosis status, and thus, L elevation mainly reflects joint inflammation in male r-axSpA patients.

**Fig. 7:**
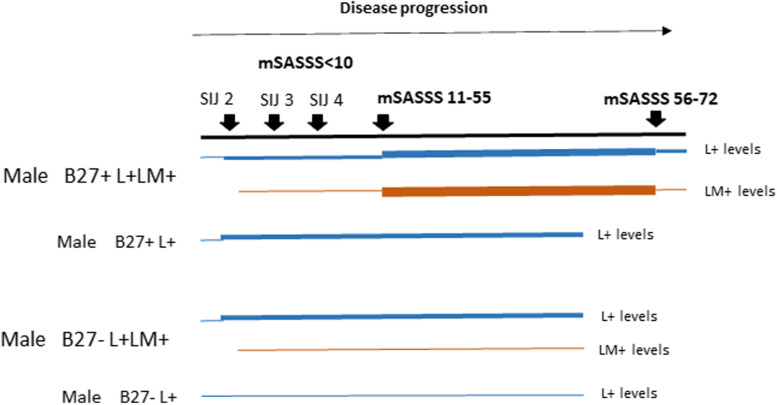
A schematics showing the differences in L+ and LM+ levels during disease progression in male r-axSpA patients with different B27 status and L/LM patterns

Elevation of LM levels is more prominent in male B27+ r-axSpA patients during spinal radiographic progression as MRI data suggests that LM levels reflect spinal inflammation more robustly than elevated L levels (Fig. 2). Figure 7 shows a schematic of this perspective. The thickness of the color bars (blue for L+ levels and orange for LM+ levels) indicates the intensities of inflammation during the disease course of male B27+ vs B27− r-axSpA patients.

## Discussion

Important findings in this report provide insight into a likely gender- and B27-related mechanism whereby spinal ankylosis progresses in male r-axSpA patients. Firstly, in axSpA patients having single LCN2 pathway involvement (i.e., OSM negative patients), similar to elevated L levels, LM levels are also significantly elevated. Our previous work using serial measurements [6] formed the basis for single-time point assessment of joint inflammation in axSpA patients. Here, for subsetting patient groups, we used 3 patterns: L+, LM+, and L+LM+, to assess their joint inflammation and ankylosis status. These patterns can be established using single-time point measurements.

Secondly, using L/LM levels from L+LM+ patients vs patients with normal L/LM levels (both subgroups have minimal spinal ankylosis), there are differences in the correlation of MRI SPARCC SIJ vs Berlin Spine Scores. Both L and LM are significantly correlated with MRI SPARCC SIJ scores, implying that both L and LM are biomarkers for SIJ inflammation, the cardinal feature of axSpA. However, using Pearson’s correlation, LM but not L is significantly correlated with MRI Berlin Spine Scores, suggesting that LM might be a better biomarker for spinal inflammation than L. Indirectly, the implication is that LM elevation might occur subsequent to L elevation, as in axSpA SIJ inflammation precedes spinal inflammation. Our observations suggest that elevated L reflects mainly SIJ inflammation and elevated LM reflects both SIJ and spinal inflammation. Thus, LM is an informative biomarker for spondylitis progression.

Thirdly, female r-axSpA patients (with minimal spinal ankylosis) had lower LM levels than their male counterparts. In addition, there are gender differences in pattern prevalence, both qualitatively and quantitatively. Male L+LM+ patients had higher L and LM levels compared to those with L+ or LM+ patterns, again implying these L+LM+ patients have more severe joint inflammation. In contrast to 65% male patients being L+LM+, 64% female patients are L+, supporting the well-known clinical observations that most male patients had more severe joint inflammation and disease progression as (1) SIJ inflammation due to both L and LM elevation and (2) spinal inflammation and progression due to LM elevation.

It is unclear why female patients have lower LM levels. In the literature, gender differences in the availability of MMP9 in target tissues have been reported [18, 19]. In axSpA, high circulating MMP levels (such as MMP9) have been associated with smoking and worse function in these patients [20].

However, it has been reported that white blood cells are the source of circulating MMP9 especially in the scenario of smoking-induce subclinical inflammatory condition [21]. Thus, serum MMP-9 levels likely do not reflect the status of MMP9 availability in the joints.

Similar to others’ reports, our cohort has a low number of female r-axSpA patients with mSASSS > 11, and thus, more female patients are needed to confirm our observations. In HC, no gender differences are observed in the prevalence of the 3 patterns (L+, LM+, and L+LM+), though very few of them (less than 10%) had elevated L/LM levels. Out of 139 HC individuals, there are only 7 L+LM+ patients (5 males and 2 females; data not shown).

Fourthly, we previously reported that higher L levels are associated with more severe ankylosis in r-axSpA patients [5]. Here, we showed that there are differences in B27+ vs B27− patients regarding the development of spinal ankylosis. In B27+ L+LM+ male patients, both L and LM levels (but not CRP) correlate with mSASS Scores: the correlation of LM levels is more robust than L levels, suggesting that both LM and L, to a lesser extent, reflect spinal inflammation and ankylosis progression. However, it is difficult to distinguish whether elevated L levels indeed reflect ankylosis progression or merely reflect inflammation at certain spinal ankylosis stages. The latter scenario is partly supported by the finding that male patients with completely fused spines (mSASSS 72) remain having fluctuating elevated L levels for years.

B27 positivity and maleness appear to have additive effects on spondylitis progression as the chances of having similarly severe diseases are lower in B27-positive female patients (Table 1). How B27 positivity leads to higher L and LM elevation especially in male patients with mSASSS > 11 (Fig. 4) remains unclear.

In B27− L+ male patients with normal LM levels, L levels correlate with mSASS Scores, suggesting elevated L alone is sufficient for disease progression, albeit at a slower rate. Although the numbers of B27− patients are low, only one out of 4 patients had mSASSS > 20 after suffering low back pain for 36 years.

**Fig. 8:**
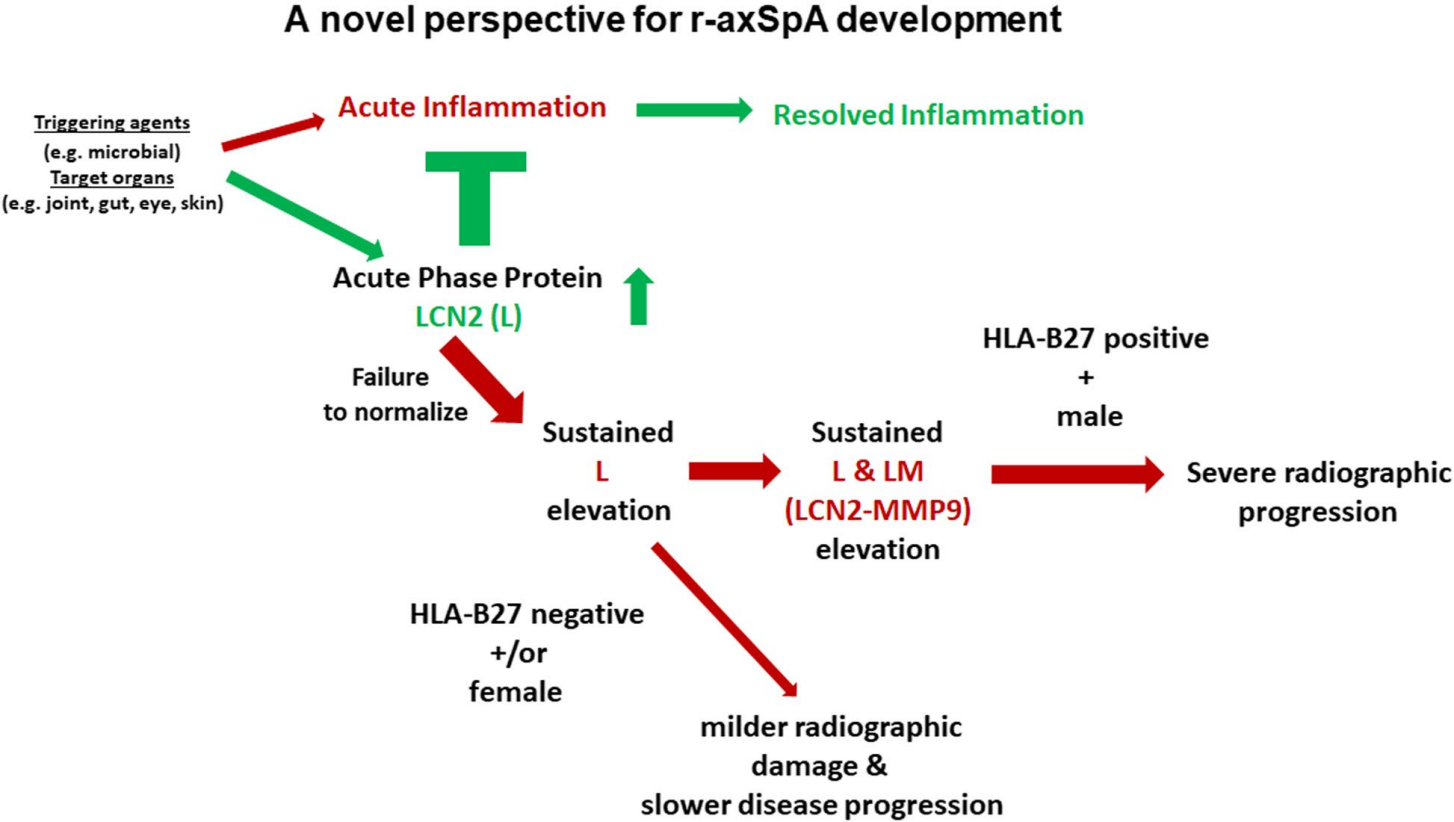
A novel perspective for r-axSpA development. LCN2 (L) as one of the acute phase proteins of the self-defense mechanism (innate immunity) is produced when triggered by agents such as microbial factors to prevent acute infection. Failure to normalize L in a timely manner would lead to chronic inflammation, reflected by elevation of L (L+) as well as concurrent elevation of L and LM (L+LM+) in the circulation. In HLA-B27-positive male patients, L+LM+ is the predominant pattern, resulting in severe SIJ and spinal inflammation and eventual drastic radiographic damage. In HLA-B27-negative male patients, and female patients (irrespective of HLA-B27 status), L+ is the predominant pattern, resulting in mainly SIJ inflammation and milder radiographic damage in the spine

We used a top-down approach to delineate steps involved in r-axSpA pathogenesis. Figure 8 summarizes our novel perspective. We previously reported that persistent elevation of L (being acute-phase proteins) resulted in chronic SIJ inflammation [6]. Here, we showed that L+LM+ B27+ male patients have a high likelihood of significant radiographic damage and progression. L+ B27− male patients and L+ female patients are more likely to have a milder disease course.

CRP is elevated in less than 30% of r-axSpA patients with active disease [22]. In this study, there is no evidence of a significant correlation of CRP with MRI or mSASS Scores, suggesting that in axSpA patients with LCN2-associated pathway involvement, CRP is not a useful biomarker.

This study focused mainly on r-axSpA male patients with involvement of a single pathway (LCN2-associated). We previously reported that 9% (27/286) of axSpA patients with involvement of only the OSM pathway have normal L and LM levels [6]. Twenty-six percent (74/286) of axSpA patients have involvement of both the LCN2-associated and OSM pathways. A preliminary analysis indicated that there are interactions between the two pathways, and in general, L levels in the OSM positive group are lower than L levels in OSM negative axSpA patients with LCN2-associated pathway (data not shown). The nature of cross-talk between the two pathways needs further investigation.

Currently, it is unclear why nr-axSpA patients do not have spinal ankylosis progression. The mechanisms appear to be different from male r-axSpA patients reported here. More studies are needed.

There are limitations in this study, the main one being relatively low sample numbers in some comparisons. It is a challenge to have adequate patient numbers as many analyses would require subgrouping of patients based on cofactors such as HLA-B27 status, gender, and comorbidities. This is the main reason why this study is focused on male r-axSpA patients. By grouping patients based on pathway involvement, homogeneous features in patient subgroups could be identified. A collaborative effort is required to have large patient cohorts for validation of our findings.

## Conclusions

Elevation of L and LM are informative biomarkers (both qualitatively and quantitatively) for SIJ and spinal inflammation, as well as for ankylosing development in r-axSpA patients. Distinctive L+LM+ or L+ patterns not only could distinguish clinically aggressive vs milder course of disease, respectively, but also provide an explanation for B27-positive male patients being highly susceptible to severe spinal ankylosis.

## Supplementary Information

**Additional file 1: Fig. S1.** Comparison of L and LM levels in male r-axSpA patients. A. L (x-axis) and LM (y-axis) levels in B27+ patients. B. L and LM levels in B27- patients. C. Both L and LM levels are significantly higher in B27+ patients compared to B27- patients. **Table S1.** Demographics of axSpA patients with LCN2-associated pathway involvement.

## Data Availability

Data sharing is not applicable to this article as no datasets were generated or analyzed during the current study.

## Abbreviations

APP: Acute phase proteins
ANOVA: One-way analysis of variance
AS: Ankylosing spondylitis
axSpA: Axial spondyloarthritis
CRP: C-reactive protein
LCN2: Lipocalin 2
Ln: Normal LCN2 level
L+: Elevation of LCN2
MRI: Magnetic resonance imaging
mSASSS: Modified Stoke Ankylosing Spondylitis Spinal Score
nr-axSpA: Non-radiographic axial spondyloarthritis
OSM: Oncostatin M
SE: Standard error
SIJ: Sacroiliac joint
SPARCC: Spondyloarthritis Research Consortium of Canada
UHN: University Health Network
95% CI: 95% confidence interval
