# Supplementary figures and images for: The role of LCN2 and LCN2-MMP9 in spondylitis radiographic development: gender and HLA-B27 status differences
Source: Arthritis Res Ther. 2022 Jul 8;24:164. doi: 10.1186/s13075-022-02854-2 (PMC9264538; doi:10.1186/s13075-022-02854-2)

**Figure S1**

**
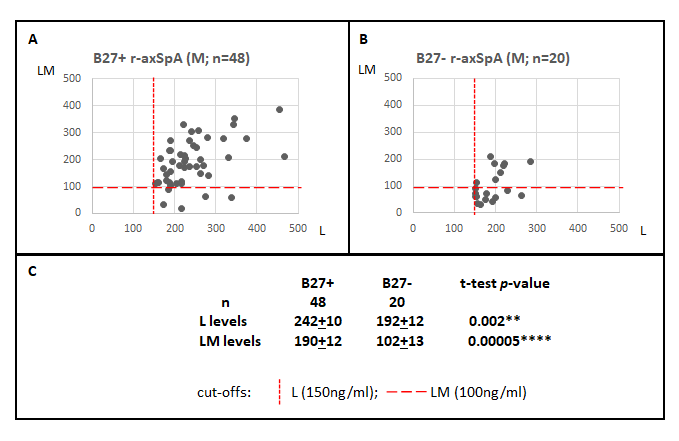
**

**Table S1**


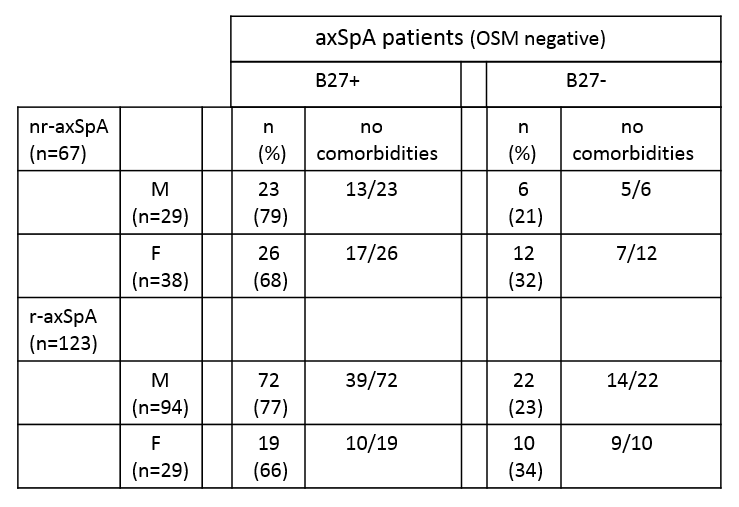

Supplement: Supplementary file 1 — Additional file 1: Fig. S1. Comparison of L and LM levels in male r-axSpA patients. A. L (x-axis) and LM (y-axis) levels in B27+ patients. B. L and LM levels in B27- patients. C. Both L and LM levels are significantly higher in B27+ patients compared to B27- patients. Table S1. Demographics of axSpA patients with LCN2-associated pathway involvement. [file 13075_2022_2854_MOESM1_ESM.docx]
